# Acquired resistance to oxaliplatin is not directly associated with increased resistance to DNA damage in SK-N-AS^r^OXALI^4000^, a newly established oxaliplatin-resistant sub-line of the neuroblastoma cell line SK-N-AS

**DOI:** 10.1371/journal.pone.0172140

**Published:** 2017-02-13

**Authors:** Emily Saintas, Liam Abrahams, Gulshan T. Ahmad, Anu-Oluwa M. Ajakaiye, Abdulaziz S. H. A. M. AlHumaidi, Candice Ashmore-Harris, Iain Clark, Usha K. Dura, Carine N. Fixmer, Chinedu Ike-Morris, Mireia Mato Prado, Danielle Mccullough, Shishir Mishra, Katia M. U. Schöler, Husne Timur, Maxwell D. C. Williamson, Markella Alatsatianos, Basma Bahsoun, Edith Blackburn, Catherine E. Hogwood, Pamela E. Lithgow, Michelle Rowe, Lyto Yiangou, Florian Rothweiler, Jindrich Cinatl, Richard Zehner, Anthony J. Baines, Michelle D. Garrett, Campbell W. Gourlay, Darren K. Griffin, William J. Gullick, Emma Hargreaves, Mark J. Howard, Daniel R. Lloyd, Jeremy S. Rossman, C. Mark Smales, Anastasios D. Tsaousis, Tobias von der Haar, Mark N. Wass, Martin Michaelis

**Affiliations:** 1 School of Biosciences, University of Kent, Canterbury, United Kingdom; 2 Industrial Biotechnology Centre, University of Kent, Canterbury, United Kingdom; 3 Institut für Medizinische Virologie, Klinikum der Goethe-Universität, Frankfurt am Main, Germany; 4 Institut für Rechtsmedizin, Klinikum der Goethe-Universität, Frankfurt am Main, Germany; University of Navarra, SPAIN

## Abstract

The formation of acquired drug resistance is a major reason for the failure of anti-cancer therapies after initial response. Here, we introduce a novel model of acquired oxaliplatin resistance, a sub-line of the non-MYCN-amplified neuroblastoma cell line SK-N-AS that was adapted to growth in the presence of 4000 ng/mL oxaliplatin (SK-N-AS^r^OXALI^4000^). SK-N-AS^r^OXALI^4000^ cells displayed enhanced chromosomal aberrations compared to SK-N-AS, as indicated by 24-chromosome fluorescence *in situ* hybridisation. Moreover, SK-N-AS^r^OXALI^4000^ cells were resistant not only to oxaliplatin but also to the two other commonly used anti-cancer platinum agents cisplatin and carboplatin. SK-N-AS^r^OXALI^4000^ cells exhibited a stable resistance phenotype that was not affected by culturing the cells for 10 weeks in the absence of oxaliplatin. Interestingly, SK-N-AS^r^OXALI^4000^ cells showed no cross resistance to gemcitabine and increased sensitivity to doxorubicin and UVC radiation, alternative treatments that like platinum drugs target DNA integrity. Notably, UVC-induced DNA damage is thought to be predominantly repaired by nucleotide excision repair and nucleotide excision repair has been described as the main oxaliplatin-induced DNA damage repair system. SK-N-AS^r^OXALI^4000^ cells were also more sensitive to lysis by influenza A virus, a candidate for oncolytic therapy, than SK-N-AS cells. In conclusion, we introduce a novel oxaliplatin resistance model. The oxaliplatin resistance mechanisms in SK-N-AS^r^OXALI^4000^ cells appear to be complex and not to directly depend on enhanced DNA repair capacity. Models of oxaliplatin resistance are of particular relevance since research on platinum drugs has so far predominantly focused on cisplatin and carboplatin.

## Introduction

Despite continuous progress over past decades, the prognosis for cancer patients whose disease cannot be controlled locally remains generally unsatisfactory. More than 90% of cancer-associated deaths occur in patients with metastatic disease and the five-year survival rates are below 20% for this group [1,2].

Effective systemic therapies are needed to improve treatment outcome. A major obstacle in the development of such therapies is the occurrence of drug resistance. Cancer cell drug resistance can be intrinsic, i.e. there is no initial therapy response in previously untreated patients, or acquired, i.e. tumours initially respond to therapy but eventually become resistant resulting in treatment failure [3]. Acquired resistance is a major problem in a wide range of cancer types [3]. An improved understanding of the processes underlying resistance acquisition is needed to develop improved therapies. Drug-adapted cancer cell lines are preclinical model systems that are used to study resistance formation in cancer cells and that have been shown to reflect clinical mechanisms of acquired resistance [4–9].

Neuroblastoma is the most frequent solid extracranial paediatric cancer entity. About half of the patients are diagnosed with high-risk disease associated with overall survival rates below 50% despite myeloablative therapy and differentiation therapy using retinoids [10–12]. Resistance acquisition is a major issue in high-risk neuroblastoma. About half of high-risk neuroblastoma patients will relapse after completion of initial therapy leaving them with survival rates below 10% [11,12]. High-risk neuroblastoma disease can be further classified into tumours with or without MYCN amplification that differ substantially in biology and therapy response [10–15].

An initial study has suggested oxaliplatin to be active in neuroblastoma cell lines [16]. Although there is limited evidence on the clinical efficacy of oxaliplatin in neuroblastoma patients, oxaliplatin has been shown to be associated with an acceptable safety profile and is suggested to display activity in some studies [17–20]. Here, we introduce a novel sub-line of the neuroblastoma cell line SK-N-AS with acquired resistance to oxaliplatin (SK-N-AS^r^OXALI^4000^). SK-N-AS was established from a bone marrow metastasis of a 6 year old female patient with non-MYCN-amplified neuroblastoma (www.atcc.org) [21].

## Materials and methods

### Cells

The non-MYCN-amplified neuroblastoma cell line SK-N-AS was obtained from ATCC (Manassas, VA, US). The oxaliplatin-resistant SK-N-AS sub-line SK-N-AS^r^OXALI^4000^ adapted to growth in the presence of oxaliplatin 4000 ng/mL was derived from the resistant cancer cell line (RCCL) collection (www.kent.ac.uk/stms/cmp/RCCL/RCCLabout.html) and had been established by previously described methods [22]. In addition, we used an SK-N-AS^r^OXALI^4000^ sub-line that had been cultivated for at least 10 passages in the absence of oxaliplatin (SK-N-AS^r^OXALI^4000(-)^) as a control. The MYCN-amplified UKF-NB-3 neuroblastoma cell line was established from bone marrow metastases of a stage IV neuroblastoma patient [23].

All cells were propagated in IMDM supplemented with 10% FBS, 100 IU/ml penicillin and 100 μg/ml streptomycin at 37°C. Cells were routinely tested for mycoplasma contamination. Authentication was performed by short tandem repeat (STR) profiling. DNA was isolated using the QIAamp DNA Blood Mini Kit (Qiagen, Hilden, Germany), and the STR analysis was performed using the PowerPlex 16 System (Promega, Mannheim, Germany) according to the manufacturers' protocols.

### Viability assay

Cell viability was tested by the 3-(4,5-dimethylthiazol-2-yl)-2,5-diphenyltetrazolium bromide (MTT) dye reduction assay after 120 h incubation modified as described previously [22].

### Virus infection

1500 viable cells were incubated for 4 days in 96-well plates prior to infection with H1N1 strain A/WSN/33 at the indicated multiplicities of infection (MOIs) as previously described previously [24]. After 48h of incubation at 37°C / 5% CO_2_ cell viability was determined by MTT assay.

### 24-chromosome fluorescence in situ hybridisation (FISH)

24-chromosome FISH was performed as described previously [25,26] using a protocol that involved six spectrally distinct fluorochromes PlatinumBright: 405 (blue), 415 (light blue/aqua), 495 (green), 547 (light red/orange), 590 (dark red), 647 (far red) plus the DAPI counterstain in a four-stage probing and re-probing strategy [25,26]. Kreatech Diagnostics synthesised all probes for this protocol, including 18 centromeric targets and six unique sequence targets for chromosomes 5, 13, 14, 19, 21 and 22 using their Universal Linkage Labelling System www.kreatech.com/rest/products/repeat-freetm-poseidontm-fish-dna-probes/preimplantation-genetic-screening/multistar-24-fish.html. Blastomere nuclei were fixed to glass slides using standard protocols described previously [27]. Slides were washed in PBS (2 min) followed by dehydration and air-drying using an ethanol series. Pepsin treatment followed (1 mg/ml pepsin in 0.01 M HCl, 20 min at 37°C), then rinsing in distilled water and PBS, then a paraformaldehyde (1% in PBS) fixation at 4°C for 10 min, then PBS and distilled water washes followed by ethanol dehydration and air drying. Four probe combinations [25] dissolved in hybridisation mix (Kreatech) were pre-denatured at 72–73°C for 10 min and pipetted on to the slide. Co-denaturation of probe and target cells at 75°C for 90 s (Thermobrite-StatSpin, Vysis/Abbott) proceeded before hybridisation at 37°C. The hybridisation period for the first three rounds of hybridisation (centromeric probes) was 30 min, whereas for the final round was overnight. Post-hybridisation washes were for 1 min 30 s in 0.7 × SSC, 0.3% Tween 20 at 72°C followed by a 2 min in 2× SSC at room temperature. Slides were mounted in Vectashield containing 0.1 ng/μL of DAPI (Vector labs) before microscopy and image analysis. After analysis and image capture, slides were washed in 2× SSC at room temperature to remove the coverslip and then washed for 30 s in distilled water (72°C) to remove the bound probe. An ethanol series preceded air-drying before continuation to the next round of hybridisation. The protocol was the same for the second, third and final rounds with the following exceptions: The overnight hybridisation time for the final round (previously mentioned), pepsin and paraformaldehyde treatment were only required for the first round; the post-hybridisation wash time was reduced with every round from 90 s (first round of hybridisation) to 50–60 s (second round) to 30 s (third and final rounds). Microscopy analysis was performed on an Olympus BX-61 epifluorescence microscope equipped with a cooled CCD camera (by Digital Scientific—Hamamatsu Orca-ER C4742-80) using the appropriate filters. To enable analysis of the fluorochromes for image acquisition two communicating filter wheels (Digital Scientific UK) with the appropriate filters were used. The recommended filters by the probe manufacturers can be found here: www.kreatech.com/rest/customer-service-support/technical-support/fluorophores-and-filter-recommendation.html

A modified version of SmartCapture software (Digital Scientific UK) was used to capture all images, and to display results from all four hybridisation rounds in the same image, Adobe Photoshop was used.

### Receptor tyrosine kinase phosphorylation array

The phosphorylation status of 49 receptor tyrosine kinases was determined using a commercial kit (Proteome Profiler Human Phospho-RTK Array Kit, R&D Systems, Abingdon, UK) following the manufacturer’s instructions. Spot sizes were examined visually and densitometric analysis was performed using ImageJ software (http://imagej.nih.gov/ij/).

### Respirometry

Oxygen consumption of intact cells was determined using two chambered Oxygraph-2k high resolution respirometer (Oroboros, Innsbruck, Austria) at 37°C in response to modulators of oxidative phosphorylation. Results were analysed using DatLab software (Oroboros, Innsbruck, Austria). Baseline respiratory activity was determined for 30 min. Then, 8 μg/mL oligomycin (Sigma-Aldrich Company Ltd, Dorset, UK) was added for 10 min. Oligomycin inhibits ATP synthase resulting in proton leak and, in turn, a leak of respiration. This was followed by treatment with FCCP (Carbonyl cyanide-4-(trifluoromethoxy) phenylhydrazone) (Sigma-Aldrich Company Ltd, Dorset, UK) 10 μM. FCCP interferes with the proton gradient by uncoupling the electron transport chain from the oxidative phosphorylation system resulting in maximal capacity of the respiratory chain.

### Cell sensitivity to Ultraviolet C (UVC)-induced DNA damage

Effects of ultraviolet C (UVC)-irradiation on cell viability were determined by MTT assay 120h post irradiation with a UVG-11 compact UV lamp (UVP, Upland, CA) at a wave length of 254 nm. Doses were calculated with a UVX digital radiometer (UVP, Upland, CA). In addition, a colony formation assay was performed. 10,000 cells were transferred into 60 mm dishes and incubated for 11 days. Colonies were fixed, stained using crystal violet solution, and counted.

### Statistics

Results are expressed as mean ± S.D. of at least three experiments. In general, comparisons between two groups were performed using Student’s t-test. Three or more groups were compared by ANOVA followed by the Student-Newman-Keuls test. The fraction of diploid cells in the project cell lines were compared by χ^2^ test with subsequent Bonferroni correction. P values lower than 0.05 were considered to be significant.

## Results

### Cell line authentication by STR analysis

Prior to the start of the project, the identity of SK-N-AS and SK-N-AS^r^OXALI^4000^ was confirmed by STR analysis (S1 Table).

### Cytogenetic cell line characterisation

**Fig 1:**
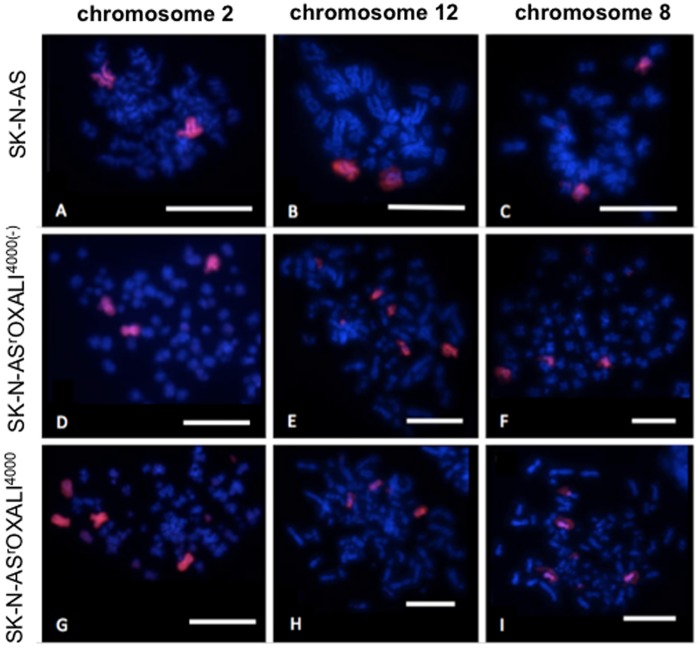
Representative fluorescence in situ hybridisation (FISH) images of chromosomes 2 (A, D and G), 12 (B, E and H) and 8 (C, F and I) in SK-N-AS (A-C), SK-N-ASrOALI4000(-) (D-F), and SK-N-ASrOXALI4000 (G-I) neuroblastoma cells. Scale bar represents 10μm.

Representative images of the cytogenetic cell line characterisation by 24-chromosome fluorescence *in situ* hybridisation (FISH) are presented in Fig 1. Aneuploidy was observed in all the cell lines. However, the level of aneuploidy overall was lower for the parental SK-N-AS cells (50% diploidy) than for the oxaliplatin-resistant SK-N-AS sub-line SK-N-AS^r^OXALI^4000^ (34% diploidy) and for SK-N-AS^r^OXALI^4000^ cells that had been cultivated for at least 10 passages in the absence of oxaliplatin (SK-N-AS^r^OXALI^4000(-)^, 38% diploidy) (S2 Table). Statistical analysis revealed significant differences in the fraction of diploid cells between SK-N-AS and SK-N-AS^r^OXALI^4000^ as well as between SK-N-AS and SK-N-AS^r^OXALI^4000(-)^ cells, but not between SK-N-AS^r^OXALI^4000^ and SK-N-AS^r^OXALI^4000(-)^ cells. Chromosome numbers ranged from 23–160 for SK-N-AS and SK-N-AS^r^OXALI^4000(-)^ cells and from 23–178 in SK-N-AS^r^OXALI^4000^ cells. The increase in chromosome number was caused by increases in specific chromosomes not whole metaphase duplication. Chromosomes 18 and X were more likely to remain diploid (50–59% and 72–95% diploidy observed respectively) versus other chromosomes that showed higher levels of variation (S2 Table).

Chromosomal rearrangement within the metaphases was also common for all cell lines investigated (S3 Table). Single colour chromosome painting of chromosomes 1, 3, 4, 5, 6, 7, 8, 13, 14, 17, 18, 22 and X showed signals on multiple chromosomes in 66–100% of metaphases analysed. Chromosomes 10, 11, 12, 15 and 21 displayed signal on a single chromosome for SK-N-AS (70–94%), but signals on multiple chromosomes for SK-N-AS^r^OXALI^4000^ and SK-N-AS^r^OXALI^4000(-)^ cells (44–100%) (S3 Table). Fig 1 shows an example of whole chromosome painting from this study. In this example, SK-N-AS cells display two signals for the chromosomes 2, 8, and 12 showing that they are diploid for these chromosomes (Fig 1A–1C). In contrast, more than two signals (Fig 1D–1I) as well as partially labelled chromosomes (Fig 1E and 1I) can be seen in SK-N-AS^r^OXALI^4000^ and SK-N-AS^r^OXALI^4000(-)^ cells.

### Cross-resistance profiles of SK-N-AS and its sub-lines

Next, we determined the drug resistance profiles of SK-N-AS, SK-N-AS^r^OXALI^4000^, and SK-N-AS^r^OXALI^4000(-)^ to a range of anti-cancer agents. This included the frequently used platinum drugs oxaliplatin, cisplatin, and carboplatin. In addition, we examined the effects of two additional anti-cancer drugs that target DNA integrity. Doxorubicin is an anthracycline that targets DNA integrity through DNA intercalation and topoisomerase II inhibition [28,29]. Gemcitabine is a nucleoside analogue that is intracellularly activated through phosphorylation [30]. Gemcitabine triphosphate is incorporated by DNA polymerase into the DNA resulting in chain termination after addition of one more nucleotide triphosphate (masked chain termination). In addition, gemcitabine diphosphate covalently binds to and inhibits ribonucleotide reductase resulting in the reduction of cellular nucleotide triphosphate pools and inhibition of deoxycytidylate deaminase that inactivates gemcitabine monophosphate. These events increase gemcitabine phosphorylation and incorporation into DNA (gemcitabine self-potentiation) [30].

**Fig 2:**
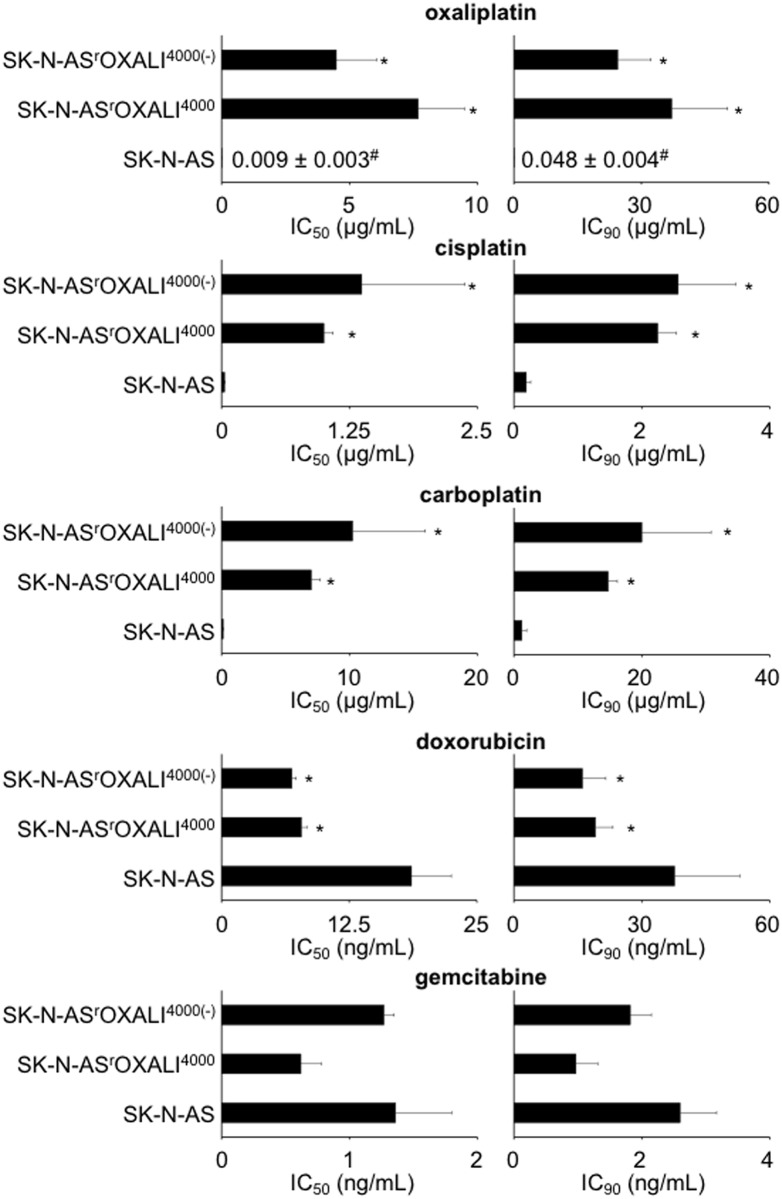
Effects of cytotoxic drugs on the viability of SK-N-AS cells, SK-N-AS cells with acquired resistance to oxaliplatin (SK-N-AS^r^OXALI^4000^), or SK-N-AS^r^OXALI^4000^ cells that had been cultivated for 10 weeks in the absence of oxaliplatin (SK-N-AS^r^OXALI^4000(-)^). Drug concentrations that reduce cell viability by 50% (IC_50_) or 90% (IC_90_) were determined by MTT assay after 120h of incubation. * P < 0.05 relative to control; ^#^ mean ± S.D. (presented when no bar is visible on the chosen scale).

The oxaliplatin IC_50_ of SK-N-AS^r^OXALI^4000^ cells was increased by 834-fold compared to SK-N-AS cells, the IC_90_ was increased by 774-fold (Fig 2). SK-N-AS^r^OXALI^4000(-)^ cells (that had been cultivated for 10 weeks in the absence of oxaliplatin) displayed slightly decreased IC_50_ and IC_90_ values compared to SK-N-AS^r^OXALI^4000^ cells but remained highly oxaliplatin-resistant (IC_50_ SK-N-AS^r^OXALI^4000(-)^/ IC_50_ SK-N-AS = 487; IC_90_ SK-N-AS^r^OXALI^4000(-)^/ IC_90_ SK-N-AS = 509) indicating a stable oxaliplatin resistance phenotype that does not depend on the continuous presence of oxaliplatin (Fig 2). SK-N-AS^r^OXALI^4000^ and SK-N-AS^r^OXALI^4000(-)^ cells displayed similar cross-resistance to cisplatin and carboplatin. The resistance factors for cisplatin and carboplatin were lower than for oxaliplatin (S4 Table).

In contrast to the cross-resistance profiles against platinum drugs, SK-N-AS^r^OXALI^4000^ and SK-N-AS^r^OXALI^4000(-)^ cells displayed no cross-resistance to doxorubicin and gemcitabine (Fig 2, S4 Table). Notably, SK-N-AS^r^OXALI^4000^ and SK-N-AS^r^OXALI^4000(-)^ cells were more sensitive to doxorubicin than SK-N-AS cells.

Differentiation therapy using retinoic acids is a common constituent of treatment protocols for high-risk neuroblastoma patients [11,12]. SK-N-AS cells were previously reported to be insensitive to all-trans retinoic acid (ATRA)-induced differentiation [31]. In concordance, neither SK-N-AS cells nor SK-N-AS^r^OXALI^4000^ or SK-N-AS^r^OXALI^4000(-)^ cells were sensitive to ATRA in concentration of up to 5μM after treatment for up to five days.

**Fig 3:**
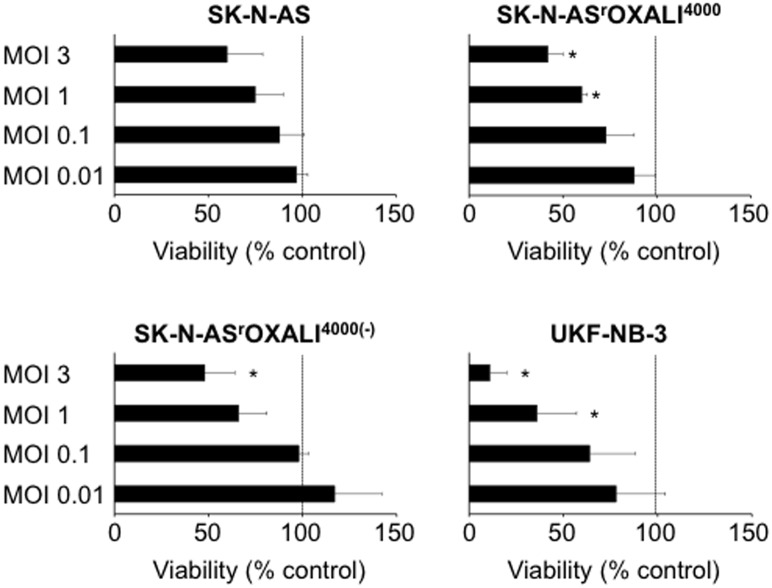
Effects of H1N1 influenza A virus infection on cell viability. Non-MYCN-amplified SK-N-AS neuroblastoma cells, SK-N-AS cells with acquired resistance to oxaliplatin (SK-N-AS^r^OXALI^4000^), SK-N-AS^r^OXALI^4000^ cells that were passaged for 10 passages in absence of oxaliplatin (SK-N-AS^r^OXALI^4000(-)^), or MYCN-amplified UKF-NB-3 neuroblastoma cells were infected with H1N1 influenza strain A/WSN/33 virus at different multiplicities of infection (MOIs) and cell viability was determined 48h post infection relative to non-treated control. The dotted line indicates the viability of non-infected control cells. * P < 0.05 relative to non-infected control cells.

Influenza A viruses are under investigation as oncolytic viruses [32–36]. To determine the permissiveness of the investigated cell lines to influenza A virus infection, they were infected with the H1N1 strain A/WSN/33 at different multiplicities of infection (MOIs). Cell viability was determined at 48h post infection by MTT assay. Influenza A virus infection did not result in a significant reduction of SK-N-AS cell viability at the investigated MOIs up to 3 (Fig 3). In contrast, influenza A virus infection significantly reduced SK-N-AS^r^OXALI^4000^ cell viability at MOIs ranging 1 and 3 and SK-N-AS^r^OXALI^4000(-)^ viability at MOI 3. Notably, MYCN-amplified UKF-NB-3 cells were found to be substantially more sensitive to influenza A virus-induced anti-cancer effects than MYCN-non-amplified SK-N-AS cells (Fig 3).

### Receptor tyrosine kinase phosphorylation

**Fig 4:**
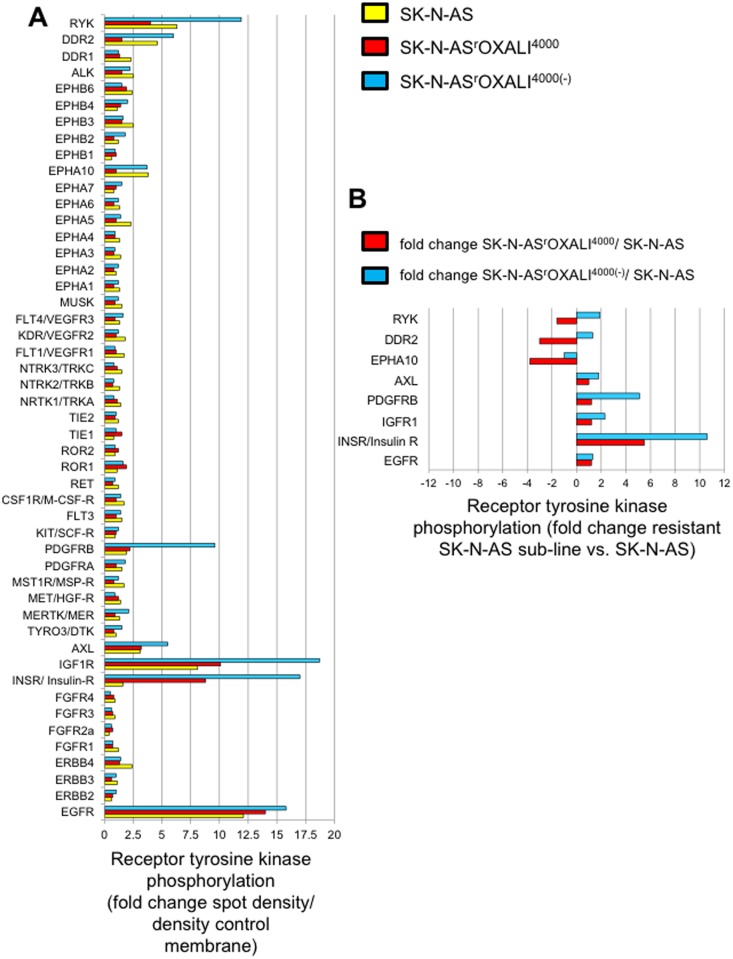
Phosphorylation status of 49 receptor tyrosine kinases. Receptor tyrosine kinase phosphorylation was determined by a commercial kit (Proteome Profiler Human Phospho-RTK Array Kit, R&D Systems, Abingdon, UK) with subsequent densitometric analysis using ImageJ software (http://imagej.nih.gov/ij/). A) Receptor tyrosine kinase phosphorylation status expressed as fold change spot density relative to a control membrane area. Images of the membranes are presented in S1 Fig. B) Differential phosphorylation of receptor tyrosine kinases that were found phosphorylated in at least one cell line (as indicated by a fold change spot density relative to a control membrane area >2) in SK-N-AS^r^OXALI^4000^ or SK-N-AS^r^OXALI^4000(-)^ cells relative to SK-N-AS.

49 receptor tyrosine kinases were analysed for their phosphorylation status (indicating kinase activation) in the project cell lines using a commercial kit (Proteome Profiler Human Phospho-RTK Array Kit, Abingdon, UK). Phosphorylation status was determined visually and densitometrically using ImageJ software. Receptor tyrosine kinases were scored as phosphorylated when spots were visible and the fold change spot density/ density control membrane was greater than 2.5. Eight receptor tyrosine kinases were phosphorylated in at least one cell line: EGFR, INSR, IGF1R, PDGFRB, AXL, EPHA10, DDR2, and RYK (Fig 4A, S1 Fig). DDR2 and EPHA10 phosphorylation was found > 2-fold reduced in SK-N-AS^r^OXALI^4000^ cells vs. SK-N-AS cells but not in SK-N-AS^r^OXALI^4000(-)^ cells vs. SK-N-AS cells suggesting that this may be a consequence of the presence of oxaliplatin. PDGFRB and IGF1R displayed increased phosphorylation in SK-N-AS^r^OXALI^4000(-)^ cells vs. SK-N-AS cells but not in SK-N-AS^r^OXALI^4000^ cells vs. SK-N-AS cells. INSR phosphorylation was > 2-fold enhanced in both SK-N-AS^r^OXALI^4000^ cells and SK-N-AS^r^OXALI^4000(-)^ cells compared to SK-N-AS cells (Fig 4B). EGFR, AXL, and RYK displayed similar phosphorylation levels in all three cell lines as indicated by fold changes of smaller than 2 (Fig 4B).

### Cellular oxygen consumption

**Fig 5:**
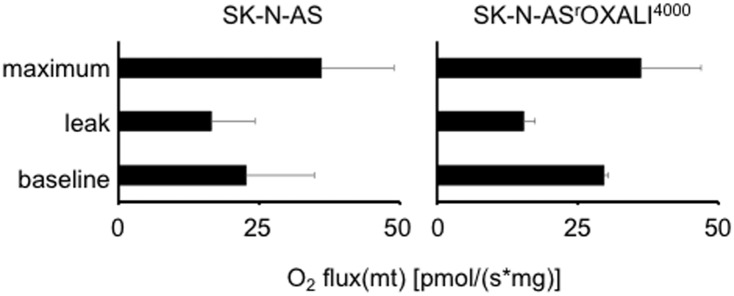
Oxygen consumption by SK-N-AS and SK-N-AS^r^OXALI^4000^ cells. Oxygen consumption was determined in intact cells in the absence of treatment (baseline), in response to oligomycin (8 μg/mL), an inhibitor of ATP synthase that causes a leak of protons resulting in inhibition of respiration (leak), and in response to FCCP (10 μM) that uncouples the electron transport chain resulting in maximum oxidative phosphorylation.

Respirometry experiments indicated that SK-N-AS and SK-N-AS^r^OXALI^4000^ cells display functional oxidative phosphorylation and mitochondria. Oligomycin reduced oxygen consumption and FCCP enhanced oxygen consumption (Fig 5). Hence, these cell lines do not appear to display a Warburg metabolism. Since there were no significant differences between the two cell lines, resistance formation to oxaliplatin does not appear to be associated with changes in the cancer cell metabolism in this model.

### Cell sensitivity to ultraviolet C (UVC)-induced DNA damage

**Fig 6:**
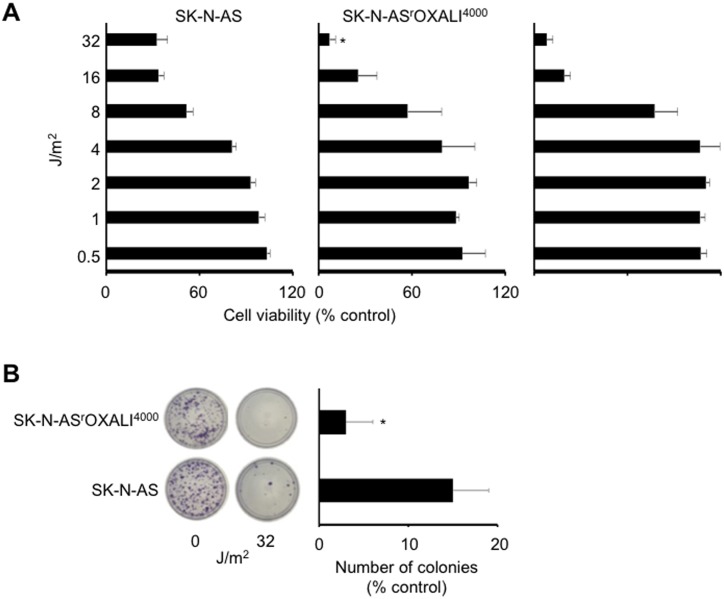
Effects of ultraviolet C (UVC) radiation on the viability of SK-N-AS and SK-N-AS^r^OXALI^4000^ cells. A) Dose-dependent effects of UVC on SK-N-AS and SK-N-AS^r^OXALI^4000^ cells as indicated by MTT assay five days post exposure. B) Representative images and quantification of colony formation by SK-N-AS and SK-N-AS^r^OXALI^4000^ cells, as determined 11 days post exposure to UVC (32 J/m^2^) relative to non-irradiated control.

Nucleotide excision repair is assumed to be critically involved in the repair of platinum drug-induced DNA damage and the nucleotide excision repair capacity has been suggested to determine cellular sensitivity to platinum drugs [37]. UVC also induces photoproducts that are exclusively repaired by nucleotide excision repair [38]. Hence, we compared the UVC response of SK-N-AS and SK-N-AS^r^OXALI^4000^ cells. Notably, SK-N-AS^r^OXALI^4000^ cells demonstrated an increased UVC sensitivity compared to SK-N-AS at higher radiation doses as indicated by MTT assay (Fig 6A). Similar results to those obtained for SK-N-AS^r^OXALI^4000^ cells were received for SK-N-AS^r^OXALI^4000^ cells (S2 Fig). This finding was confirmed by colony formation assay (Fig 6B). These results do not suggest a contribution of increased nucleotide excision repair as being relevant to the oxaliplatin resistance mechanism in SK-N-AS^r^OXALI^4000^ cells.

## Discussion

Here, we introduce SK-N-AS^r^OXALI^4000^, a sub-line of the non-MYCN-amplified neuroblastoma cell line SK-N-AS with acquired resistance to oxaliplatin. SK-N-AS^r^OXALI^4000^ cells displayed pronounced oxaliplatin resistance compared to parental SK-N-AS cells. Some studies have reported that the formation of resistance to anti-cancer drugs may be the consequence of a reversible enrichment of a pre-existing fraction of resistant cancer cells [39–41]. However, SK-N-AS^r^OXALI^4000^ cells displayed a stable resistance phenotype that was maintained after a cultivation period of 10 passages in the absence of oxaliplatin.

Cytogenetic cell line characterisation demonstrated that SK-N-AS^r^OXALI^4000^ cells acquired additional chromosomal aberrations in addition to those observed in SK-N-AS cells as indicated by increased levels of aneuploidy and chromosomal rearrangements. This may not be unexpected given that platinum drugs including oxaliplatin are thought to exert their anti-cancer effects predominantly via causing DNA damage [42,43].

Previously, it was shown that a Warburg metabolism, i.e. the use of glycolysis for ATP production instead of mitochondrial oxidative phosphorylation in the presence of sufficient oxygen levels (aerobic glycolysis), may protect cancer cells from toxicity induced by platinum drugs including oxaliplatin [44,45]. However, respirometry experiments indicated similarly intact oxidative phosphorylation in both SK-N-AS and SK-N-AS^r^OXALI^4000^ cells indicating no there was no metabolic shift towards a Warburg phenotype in oxaliplatin resistance.

Of the 49 receptor kinases examined, five kinases were found to be differentially phosphorylated between the parental SK-N-AS cell line and its oxaliplatin-resistant sub-line. DDR2 and EPHA10 phosphorylation were found to be reduced in SK-N-AS^r^OXALI^4000^ cells vs. SK-N-AS cells but not in SK-N-AS^r^OXALI^4000(-)^ cells vs. SK-N-AS cells. This suggests that these changes may be a consequence of the continuous presence of oxaliplatin during cell cultivation in SK-N-AS^r^OXALI^4000^ cells, rather than a sustainable resistance mechanism. Little is known about a role of DDR2 or EPHA10 in the cellular response to platinum drugs. DDR2 is an extracellular matrix receptor that may contribute to oncogenic processes, in particular through interaction with collagen [46]. EPHA10 is recognised as potential therapeutic target in breast cancer [47], but its functional role remains to be characterised.

PDGFRB and IGF1R displayed increased phosphorylation in SK-N-AS^r^OXALI^4000(-)^ cells vs. SK-N-AS cells but not in SK-N-AS^r^OXALI^4000^ cells vs. SK-N-AS cells. Although PDGFRB and IGF1R are well known anti-cancer drug targets previously shown to be involved in platinum drug resistance [48,49], it remains unclear how increased phosphorylation, which was exclusively observed in oxaliplatin-resistant cells that had been cultivated in the absence of oxaliplatin, may specifically contribute to oxaliplatin resistance in SK-N-AS^r^OXALI^4000^ cells. Interestingly, cisplatin has been described to reduce PDGFRB expression in neuroblastoma cells [50]. Hence, the difference between the PDGFRB levels in SK-N-AS^r^OXALI^4000^ and SK-N-AS^r^OXALI^4000(-)^ cells may be again the consequence of the presence of oxaliplatin during the culturing of SK-N-AS^r^OXALI^4000^ cells.

INSR phosphorylation was consistently increased in both SK-N-AS^r^OXALI^4000^ cells and SK-N-AS^r^OXALI^4000(-)^ cells compared to SK-N-AS cells. This may suggest a role of INSR in the context of acquired oxaliplatin resistance. However, INSR signalling has so far been associated with adverse events that have limited the clinical success of anti-cancer strategies targeting IGF1R [51]. Therefore, the relevance of INSR phosphorylation remains unclear in the context of acquired oxaliplatin resistance in our model.

Oxaliplatin belongs to the class of platinum-based anti-cancer drugs that target DNA integrity through direct chemical interaction with DNA resulting in DNA strand breaks. In addition to oxaliplatin, cisplatin and carboplatin are platinum-based drugs that are frequently used in cancer patients. Based on the analysis of drug-DNA adducts, the mechanisms of action of cisplatin and carboplatin appear to be very similar while oxaliplatin differs in its mode of action from these two compounds [42,43]. Moreover, oxaliplatin may offer a more favourable toxicity profile compared to the other frequently used platinum-based anti-cancer agents, cisplatin and carboplatin, and the cross-resistance profiles between oxaliplatin and cisplatin/ carboplatin may be incomplete [42,43,52–55]. In this context, SK-N-AS^r^OXALI^4000^ cells displayed substantial cross-resistance to cisplatin and carboplatin although the oxaliplatin resistance was much more pronounced.

Interestingly, SK-N-AS^r^OXALI^4000^ cells did not show cross-resistance to doxorubicin and gemcitabine that induce DNA damage by alternative mechanisms [28–30]. While the gemcitabine sensitivity was similar in SK-N-AS and SK-N-AS^r^OXALI^4000^ cells, SK-N-AS^r^OXALI^4000^ cells were more sensitive to doxorubicin compared to SK-N-AS. The reasons for this increased doxorubicin sensitivity remain unclear. Not much is known about the doxorubicin sensitivity of platinum drug-adapted cancer cell line. However, cell line adaptation to platinum drugs does not seem to be generally associated with increased doxorubicin sensitivity. A cisplatin-resistant sub-line of the neuroblastoma cell line UKF-NB-3 displayed cross-resistance to doxorubicin [56]. Among six cisplatin-adapted urothelial cancer cell lines, only one showed > 2-fold increased sensitivity to doxorubicin compared to the respective parental cell line, while the other five cell lines were similar sensitive to doxorubicin like the corresponding parental cell lines [57].

SK-N-AS^r^OXALI^4000^ cells also displayed higher sensitivity to UVC irradiation than SK-N-AS cells, although both UVC- and oxaliplatin-induced DNA damage are thought to be predominantly repaired by nucleotide excision repair [38,58]. In this context, the nucleotide excision repair capacity was suggested to determine cellular sensitivity to platinum drugs [37]. However, these findings may not be too surprising, given the well documented complexity of the mechanisms that may underlie platinum drug resistance [58,59]. This involves mechanisms that prevent platinum drug binding to DNA (pre-target resistance), mechanisms that inhibit cell death signalling downstream of DNA damage (post-target resistance), and/ or mechanisms that do not have obvious links with the mechanism of action of platinum drugs (off-target resistance) instead of, or in addition to, mechanisms that directly relate to platinum drug-induced DNA damage [58,59].

Oncolytic viruses including influenza viruses are under preclinical investigation as anti-cancer agents [32–36,60]. Virus-induced oncolytic effects have been described to be primarily caused by virus replication associated with cancer cell lysis and cell death induction [32,35,36,60]. Talimogene laherparepvec (T-VEC), an engineered herpes simplex virus-1, was recently approved for the therapy of melanoma in the US and Europe. Interestingly, SK-N-AS^r^OXALI^4000^ cells displayed increased sensitivity to influenza virus infection compared to SK-N-AS cells. The reasons for this remain unclear. The activation of different oncogenes has been described to promote virus replication in cancer cells [32,60], although it is not clear whether this may be the mechanism in SK-N-AS^r^OXALI^4000^ cells. Nevertheless, this finding indicates that resistance acquisition to anti-cancer drugs may be associated with a change in susceptibility to oncolytic viruses and that oncolytic viruses may represent treatment options for cancer diseases after therapy failure even if the primary tumour was not susceptible.

In conclusion, we introduce a novel oxaliplatin-resistant neuroblastoma cell line, SK-N-AS^r^OXALI^4000^. The oxaliplatin resistance mechanisms appear to be complex and not be directly associated to enhanced DNA repair capacity. Follow-up studies using methods including transcriptomics and genomics analyses will be needed to elucidate the resistance mechanisms in more detail. Models of oxaliplatin resistance are of particular relevance since research on platinum drugs has so far vastly been focused on cisplatin (62,748 hits in PubMed, www.ncbi.nlm.nih.gov/pubmed) and carboplatin (14,705 hits in PubMed), which are believed to share the same mechanism of action [42,43], in comparison to oxaliplatin (8360 hits in PubMed, data retrieved on 30^th^ November 2016).

## Supporting information

S1 Fig Kinome arrays of SK-N-AS, SK-N-AS^r^OXALI^4000^, and SK-N-AS^r^OXALI^4000(-)^ cells. (PDF)

S2 Fig Effects of ultraviolet C (UVC) radiation on the viability of SK-N-AS^r^OXALI^4000(-)^ cells. (PDF)

S1 Table Short tandem repeat (STR) profiles of SK-N-AS and SK-N-AS^r^OXALI^4000^. (PDF)

S2 Table Table summarising the number of chromosomes observed in metaphases in SK-N-AS, SK-N-AS^r^OXALI^4000(-)^, or SK-N-AS^r^OXALI^4000^ cells. (PDF)

S3 Table Table summarising the number of fully and partially labelled chromosomes observed in SK-N-AS, SK-N-AS^r^OXALI^4000^, or SK-N-AS^r^OXALI^4000(-)^ cells. (PDF)

S4 Table Neuroblastoma cell drug sensitivity as indicated by drug concentrations that reduce cell viability by 50% (IC_50_) or 90% (IC_90_). (PDF)
