# Supplementary material for: Acquired resistance to oxaliplatin is not directly associated with increased resistance to DNA damage in SK-N-ASrOXALI4000, a newly established oxaliplatin-resistant sub-line of the neuroblastoma cell line SK-N-AS
Source: PLoS One. 2017 Feb 13;12(2):e0172140. doi: 10.1371/journal.pone.0172140 (PMC5305101; doi:10.1371/journal.pone.0172140)
Supplement: S1 Fig — (PDF) [file pone.0172140.s001.pdf]

S1 Figure

A

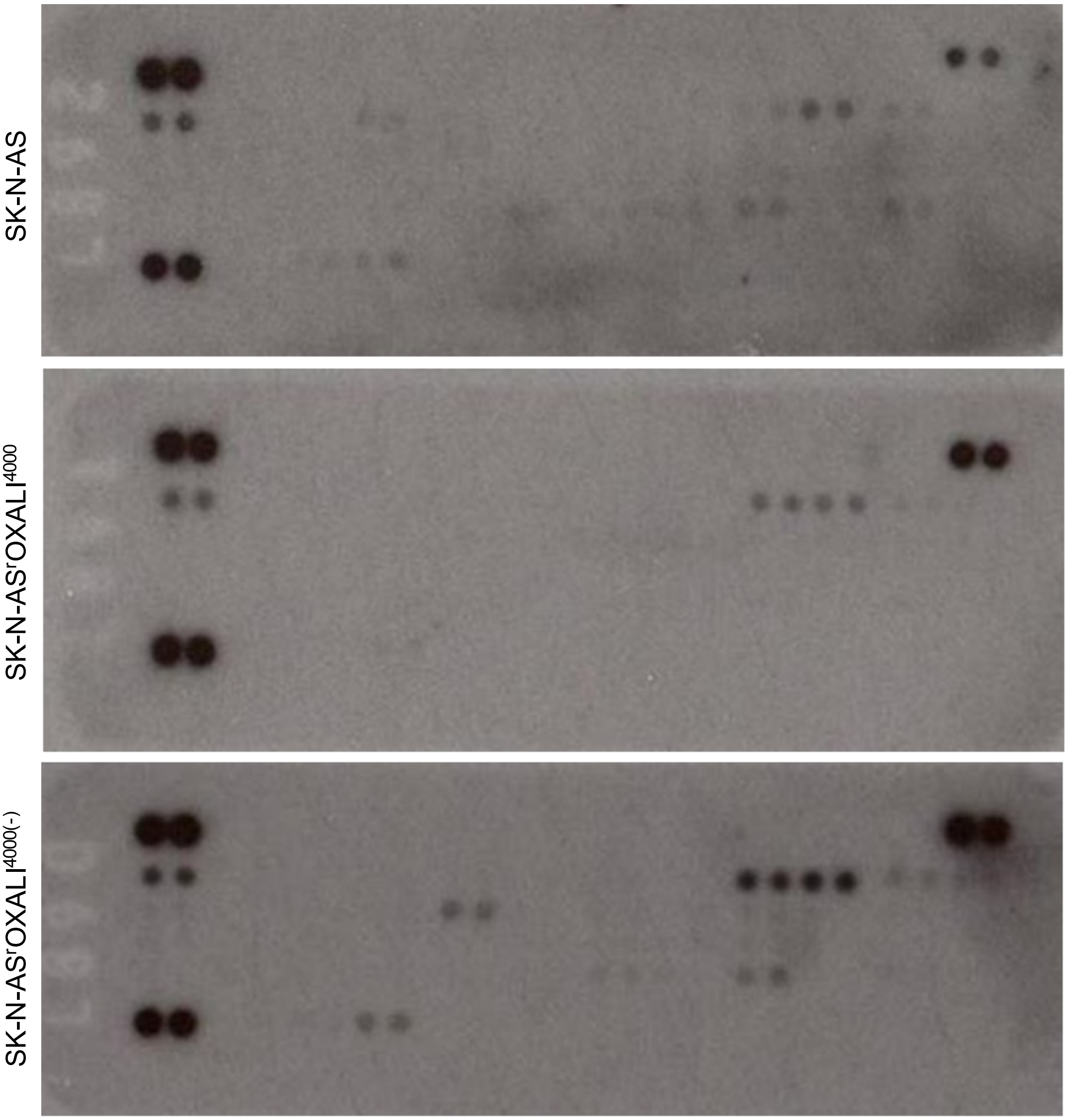

B

Human Phospho-RTK Array Coordinates

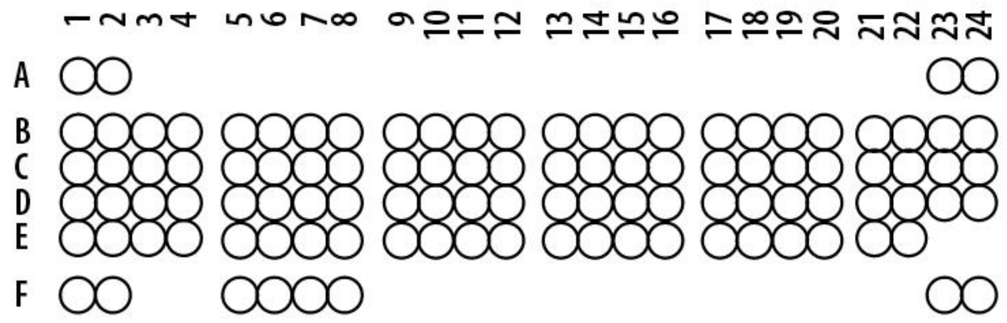

Coordinate	Receptor Family	RTK/Control	Coodinate	Receptor Family	RTK/Control
A1, A2	Reference Spots	—	D1, D2	Tie	Tie-2
A23, A24	Reference Spots	—	D3, D4	NGF R	TrkA
B1, B2	EGF R	EGF R	D5, D6	NGF R	TrkB
B3, B4	EGF R	ErbB2	D7, D8	NGF R	TrkC
B5, B6	EGF R	ErbB3	D9, D10	VEGF R	VEGF R1
B7, B8	EGF R	ErbB4	D11, D12	VEGF R	VEGF R2
B9, B10	FGF R	FGF R1	D13, D14	VEGF R	VEGF R3
B11, B12	FGF R	FGF R2α	D15, D16	MuSK	MuSK
B13, B14	FGF R	FGF R3	D17, D18	Eph R	EphA1
B15, B16	FGF R	FGF R4	D19, D20	Eph R	EphA2
B17, B18	Insulin R	Insulin R	D21, D22	Eph R	EphA3
B19, B20	Insulin R	IGF-I R	D23, D24	Eph R	EphA4
B21, B22	Axl	Axl	E1, E2	Eph R	EphA6
B23, B24	Axl	Dtk	E3, E4	Eph R	EphA7
C1, C2	Axl	Mer	E5, E6	Eph R	EphB1
C3, C4	HGF R	HGF R	E7, E8	Eph R	EphB2
C5, C6	HGF R	MSP R	E9, E10	Eph R	EphB4
C7, C8	PDGF R	PDGF Rα	E11, E12	Eph R	EphB6
C9, C10	PDGF R	PDGF Rβ	E13, E14	Insulin R	ALK
C11, C12	PDGF R	SCF R	E15, E16	—	DDR1
C13, C14	PDGF R	Flt-3	E17, E18	—	DDR2
C15, C16	PDGF R	M-CSF R	E19, E20	Eph R	EphA5
C17, C18	RET	c-Ret	E21, E22	Eph R	EphA10
C19, C20	ROR	ROR1	F1, F2	Reference Spots	—
C21, C22	ROR	ROR2	F5, F6	Eph R	EphB3
C23, C24	Tie	Tie-1	F7, F8	—	RYK
			F23, F24	Control (-)	PBS

S1 Figure. Kinome arrays of SK-N-AS, SK-N-AS^{r}OXALI^{4000}, and SK-N-AS^{r}OXALI$^{4000(-)}$ cells. A) Autoradiographs, 1.7 mg/mL of protein were added to each of the kinome arrays; B) Organisation of the kinome array.
