# Supplementary material for: Acquired resistance to oxaliplatin is not directly associated with increased resistance to DNA damage in SK-N-ASrOXALI4000, a newly established oxaliplatin-resistant sub-line of the neuroblastoma cell line SK-N-AS
Source: PLoS One. 2017 Feb 13;12(2):e0172140. doi: 10.1371/journal.pone.0172140 (PMC5305101; doi:10.1371/journal.pone.0172140)
Supplement: S2 Fig — (PDF) [file pone.0172140.s002.pdf]

S2 Figure

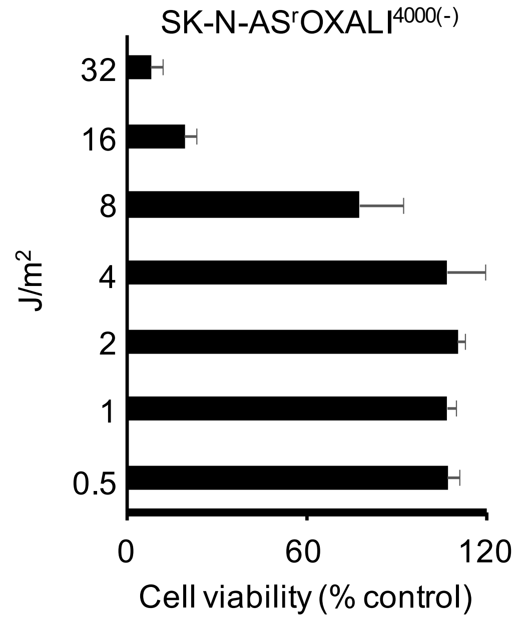

S2 Figure. Effects of ultraviolet C (UVC) radiation on the viability of SK-N-AS^{r}OXALI$^{4000(-)}$ cells. Dose-dependent effects were detected by MTT assay five days post exposure.
