# Supplementary material for: Acquired resistance to oxaliplatin is not directly associated with increased resistance to DNA damage in SK-N-ASrOXALI4000, a newly established oxaliplatin-resistant sub-line of the neuroblastoma cell line SK-N-AS
Source: PLoS One. 2017 Feb 13;12(2):e0172140. doi: 10.1371/journal.pone.0172140 (PMC5305101; doi:10.1371/journal.pone.0172140)
Supplement: S1 Table — (PDF) [file pone.0172140.s003.pdf]

S1 Table. Short tandem repeat (STR) profiles of SK-N-AS and SK-N-AS[r]OXALI[4000].

	D5 S818	D13 S317	D7 S820	D16 S539	VWA	TH 01	Amel	TPOX	CSF1 PO
SK-N-AS (reference profile)	**11/12**	**9/9**	**11/13**	**14/14**	**16/17**	**9,3/9,3**	**x/x**	**11/12**	**10/12**
SK-N-AS	11/12	9/9	11/13	14/14	16/17	9,3/9,3	x/x	11/12	10/12
SK-N-ASrOXALI4000	11/12	9/9	11/13	14/14	16/17	9,3/9,3	x/x	11/12	10/12
