# Supplementary material for: Acquired resistance to oxaliplatin is not directly associated with increased resistance to DNA damage in SK-N-ASrOXALI4000, a newly established oxaliplatin-resistant sub-line of the neuroblastoma cell line SK-N-AS
Source: PLoS One. 2017 Feb 13;12(2):e0172140. doi: 10.1371/journal.pone.0172140 (PMC5305101; doi:10.1371/journal.pone.0172140)
Supplement: S2 Table — (PDF) [file pone.0172140.s004.pdf]

S2 Table. Number of chromosomes observed in metaphases in SK-N-AS, SK-N-AS^{r}OXALI$^{4000(-)}$, or SK-N-AS^{r}OXALI^{4000} cells; Nº, number; n = 23.

Level of ploidy (number of chromosomes)	SK-N-AS	SK-N-AS^{r}OXALI$^{4000(-)}$	SK-N-AS^{r}OXALI^{4000}
	Nº metaphase	Nº metaphase	Nº metaphase
Haploidy (#23-45)	66	9	12
Diploidy (#46-68)	188	157	134
Triploidy (#69-91)	96	167	205
Tetraploidy (#92-114)	13	57	32
Pentaploidy (#115-137)	7	15	14
Hexaploidy (#138-160)	3	8	2
Heptaploidy (#161-183)	0	0	1
**Total**	373	414	400
