# Supplementary material for: Acquired resistance to oxaliplatin is not directly associated with increased resistance to DNA damage in SK-N-ASrOXALI4000, a newly established oxaliplatin-resistant sub-line of the neuroblastoma cell line SK-N-AS
Source: PLoS One. 2017 Feb 13;12(2):e0172140. doi: 10.1371/journal.pone.0172140 (PMC5305101; doi:10.1371/journal.pone.0172140)
Supplement: S3 Table — (PDF) [file pone.0172140.s005.pdf]

S3 Table. Number of fully and partially labelled chromosomes observed in the cell line SK-N-AS, its oxaliplatin-resistant sub-line SK-N-AS^{r}OXALI^{4000}, SK-N-AS^{r}OXALI^{4000} cells that had been cultivated for ≥ 10 weeks in the absence of oxaliplatin (SK-N-AS^{r}OXALI$^{4000(-)}$) line metaphases (Nº, number; n= 23).

Chromosome	SK-N-AS		SK-N-AS^{r}OXALI$^{4000(-)}$		SK-N-AS^{r}OXALI^{4000}	
	Nº metaphases (number of images signal observed/number of images viewed)		Nº metaphases with chromosomes (number of images signal observed/number of images viewed)		Nº metaphases with chromosomes (number of images signal observed/number of images viewed)	
	Fully labelled	Partially labelled	Fully labelled	Partially labelled	Fully labelled	Partially labelled
1	85 (16/16)	33 (15/16)	103 (19/19)	47 (19/19)	64 (17/17)	44 (17/17)
2	40 (9/9)	2 (1/9)	62 (17/18)	12 (5/18)	64 (16/16)	16 (8/16)
3	21 (11/13)	17 (11/13)	45 (16/17)	31 (13/17)	34 (15/16)	41 (16/16)
4	73 (18/18)	46 (18/18)	49 (16/16)	24 (15/16)	57 (16/16)	27 (13/16)
5	62 (16/16)	26 (15/16)	95 (19/19)	31 (15/19)	77 (18/18)	25 (13/18)
6	53 (17/17)	29 (13/17)	59 (15/15)	32 (13/15)	62 (19/19)	47 (18/19)
7	60 (16/16)	23 (13/16)	56 (18/18)	75 (18/18)	52 (18/18)	66 (17/18)
8	77 (17/17)	38 (12/17)	76 (16/16)	37 (12/16)	102 (18/18)	64 (18/18)

9	**59 (15/15)**	**8 (3/15)**	**76 (15/15)**	**8 (4/15)**	**61 (14/14)**	**11 (6/14)**
10	**56 (18/18)**	**2 (1/18)**	**54 (18/18)**	**71 (18/18)**	**42 (15/15)**	**58 (15/15)**
11	**38 (16/16)**	**17 (10/16)**	**38 (18/18)**	**30 (16/18)**	**33 (18/18)**	**30 (16/18)**
12	**69 (18/18)**	**9 (6/18)**	**106 (17/17)**	**31 (13/17)**	**66 (16/16)**	**42 (15/16)**
13	**40 (16/16)**	**20 (9/16)**	**59 (16/17)**	**68 (17/17)**	**54 (17/18)**	**34 (13/18)**
14	**31 (12/16)**	**26 (12/16)**	**44 (16/19)**	**47 (15/19)**	**59 (15/16)**	**26 (9/16)**
15	**58 (17/17)**	**8 (5/17)**	**86 (16/16)**	**42 (16/16)**	**86 (16/16)**	**18 (7/16)**
16	**60 (15/15)**	**0 (0/15)**	**117 (22/22)**	**1 (1/22)**	**108 (19/19)**	**0 (0/19)**
17	**56 (14/14)**	**26 (9/14)**	**75 (18/18)**	**46 (16/18)**	**58 (15/15)**	**39 (15/15)**
18	**43 (16/16)**	**21 (10/16)**	**56 (19/19)**	**72 (19/19)**	**44 (17/17)**	**43 (17/17)**
19	**42 (23/23)**	**0 (23/0)**	**66 (20/20)**	**0 (0/20)**	**82 (19/19)**	**16 (9/19)**
20	**48 (21/21)**	**1 (1/21)**	**90 (21/21)**	**1 (21/21)**	**72 (19/19)**	**0 (0/19)**
21	**51 (15/15)**	**8 (4/15)**	**54 (16/16)**	**33 (13/16)**	**96 (17/18)**	**64 (15/18)**
22	**25 (10/10)**	**11 (7/10)**	**59 (17/17)**	**48 (15/17)**	**65 (17/17)**	**45 (15/17)**
X	**31 (18/18)**	**22 (14/18)**	**35 (19/19)**	**37 (19/19)**	**37 (19/19)**	**36 (18/19)**
