# Supplementary material for: Acquired resistance to oxaliplatin is not directly associated with increased resistance to DNA damage in SK-N-ASrOXALI4000, a newly established oxaliplatin-resistant sub-line of the neuroblastoma cell line SK-N-AS
Source: PLoS One. 2017 Feb 13;12(2):e0172140. doi: 10.1371/journal.pone.0172140 (PMC5305101; doi:10.1371/journal.pone.0172140)
Supplement: S4 Table — (PDF) [file pone.0172140.s006.pdf]

S4 Table. Neuroblastoma cell drug sensitivity as indicated by drug concentrations that reduce cell viability by 50 % (IC_{50}) or 90 % (IC_{90}) determined after 120 h of incubation by MTT assay.

	SK-N-AS		SK-N-AS^{r}OXALI^{4000}		SK-N-AS^{r}OXALI$^{4000(-)}$	
drug	IC_{50}	IC_{90}	IC_{50}	IC_{90}	IC_{50}	IC_{90}
oxaliplatin (µg/mL)	0.0092 ± 0.0030	0.048 ± 0.004	7.68 ± 1.81 (835)[1]	37.1 ± 13.2 (774)	4.48 ± 1.58 (487)	24.4 ± 7.9 (509)
cisplatin (µg/mL)	0.030 ± 0.007	0.193 ± 0.070	1.00 ± 0.09 (33)	2.25 ± 0.29 (12)	1.37 ± 1.01 (46)	2.57 ± 0.90 (13)
carboplatin (µg/mL)	0.121 ± 0.020	1.24 ± 0.75	7.02 ± 0.69 (58)	14.7 ± 1.4 (12)	10.3 ± 5.7 (85)	20.0 ± 10.8 (16)
doxorubicin (ng/mL)	18.6 ± 3.9	37.7 ± 15.3	7.81 ± 0.54 (0.4)	19.1 ± 4.1 (0.5)	6.9 ± 0.4 (0.4)	16.1 ± 5.5 (0.4)
gemcitabine (ng/mL)	1.36 ± 0.44	2.60 ± 0.57	0.62 ± 0.16 (0.5)	0.97 ± 0.34 (0.4)	1.27 ± 0.08 (0.9)	1.82 ± 0.33 (0.7)

[1] fold change relative to SK-N-AS
